# Scope of Preparation of Oval and Long-Oval Root Canals: A Review of the Literature

**DOI:** 10.1155/2021/5330776

**Published:** 2021-08-23

**Authors:** Christianne Velozo, Victor Felipe Farias Prado, Ismael Sebastião da Silva Sousa, Maria Beatriz Arruda Albuquerque, Luiza Montenegro, Silmara Silva, Priscila Silva, Diana Albuquerque

**Affiliations:** Department of Restorative Dentistry and Endodontics, Dental College of Pernambuco, University of Pernambuco, Recife, Brazil

## Abstract

Successful endodontic treatment depends on cleaning and disinfecting the root canals, in order to provide conditions for three-dimensional filling, which should prevent root canal reinfection. However, anatomical complexities pose a challenge during endodontic treatment. The present study was a literature review carried out in the following databases: PubMed, SciELO, and MEDLINE, which were searched for articles published from 2017 to 2021. Micro-CT studies published in English, which analysed the capacity for preparation of oval and long-oval root canals, were included. The following keywords were used: “oval-shaped canals,” “long-oval-shaped canals,” “endodontics,” and “micro-CT.” The aim of this study was to carry out a literature review of micro-CT studies on the scope of the capacity for preparation performed in oval and long-oval root canals with rotary and reciprocating instruments.

## 1. Introduction

Chemical-mechanical preparation of root canals is recognized as being one of the most important steps in endodontic treatment [1]. The aim is to perform cleaning and disinfection and eliminate microbial irritants that are the main causes of apical periodontitis [2]. Therefore, we can affirm that the success of endodontic treatment depends on cleaning and shaping the root canal [3]. Furthermore, the kinematics and number of instruments used in the treatment can also influence the final quality of canal preparation [4].

The use of automated instrumentation in endodontics has improved the quality and predictability of preparation and significantly reduced procedural errors [5], given that heat-treated nickel-titanium (NiTi) instruments have better mechanical behaviour, flexibility, and resistance to cyclic fatigue than conventional NiTi instruments [6, 7]. The development of a new generation of nickel-titanium (NiTi) systems for root canal preparation involves characteristics such as changes in design, alloy, and kinematics, with the aim of optimizing instrumentation mechanics [7].

However, despite improvements in endodontic treatment techniques, the systems available at present still fail to completely debride the canal, especially those that have a more complex anatomy [8]. Oval and flat canals can make complete disinfection difficult, especially because they have areas that are difficult to access, favouring the accumulation of debris and microorganisms [9]. The prevalence of long-oval canals in the apical region occurs in one-third of human teeth, approximately 25%. In teeth such as mandibular incisors and maxillary second premolars, the prevalence is higher than 50%, while in the distal root of mandibular molars, it ranges from 25% to 30% [10].

Thus, mechanical preparation of the canal with a transverse oval shape can leave walls untouched which, if untreated, are filled with residual debris and/or microbial biofilm [11] that serve as a persistent source of infection [5]. Inefficient cleaning with untouched areas can result in endodontic treatment failure or delayed healing [12]. In rounded or oval root canals, being able to perform this step with the endodontic arsenal available can be stimulating work [13]. Analysis by computed microtomography (micro-CT) allows a more accurate view of the root canal than a conventional tomograph; with the use of a specific software, it is possible to accurately assess the biomechanical preparation and the anatomy, so that micro-CT is a good tool for three-dimensional and non-destructive evaluation of the root canal system [5, 6]. The aim of the present study was to carry out a literature review on the scope of preparation performed in oval and long-oval canals, with the use of rotary and reciprocating instruments.

## 2. Material and Methods

The present study was a literature review carried out in the following databases: PubMed, SciELO, and MEDLINE, which were searched for articles published from 2017 to 2021. Micro-CT studies published in English, which analysed the capacity for preparation of oval and long-oval root canals, were included. The following keywords were used: “oval-shaped canals,” “long-oval-shaped canals,” “endodontics,” and “micro-CT.” The following studies were considered eligible for the present review: (1) a microcomputed tomography study, (2) shaping ability, (3) rotary and reciprocating instruments, and (4) studies including oval-shaped canals or long-oval-shaped canals. Based on this inclusion criterion, nine articles [12–20] were selected for reading the full text.

## 3. Review

Velozo et al. [1] evaluated and compared the capacity for preparation of the XP-endo Shaper and Protaper Next instruments. Twenty mandibular incisors of long-oval canals (*n* = 10 per group) were scanned before and after root canal preparation. Parameters such as morphometric measures of volume, surface area, structure model index, and untouched walls were evaluated. The root canals were divided into two groups, according to the instrument used. By means of micro-CT analysis, no statistically significant difference was found in the percentage increase in volume (107.50%–93.13%), surface area (27.74%–29.68%), or walls untouched (13.08%–11.74%) between the XP-endo Shaper and Protaper Next groups, respectively. As regards the structure model index (SMI) parameter, the mean increase was 30.86% in the XP-endo Shaper group and 30.30% in the ProTaper Next group (*p* > 0.05).

The cleaning and shaping capability of four instrument systems, WaveOne Gold, TRUShape, EdgeCoil, and XP-endo Shaper, was investigated by Thomas et al. [15]. Thirty-two mandibular premolars with oval canals were scanned before and after root canal preparation. Aspects such as surface area, volume, SMI, and percentage of untouched walls were evaluated. The micro-CT analysis showed an increase in volume of approximately 22.44%, 19.69%, 27.31%, and 17.71%; surface area 19.65%, 14.05%, 24.34%, and 14.23%; untouched walls 50.09%, 55.26%, 38.09%, and 52.08%; and 0.91%, 3.13%, 4.06%, and 2.48% for the SMI parameter, when performing the preparation with WaveOne Gold, TRUShape, EdgeCoil, and XP-endo Shaper, respectively. No system was capable of touching the entire root canal area; the only outstanding performance found was that of EdgeCoil that achieved the highest increase in values of volume and surface area, as well as the smallest quantity of untouched walls, among all the systems tested.

In a micro-CT study, Xavier et al. [16] evaluated the capacity for preparation of forty canines with oval canals (*n* = 20), performed with XP-endo Shaper and Mtwo systems. Image processing of the entire length of the canal and apical third (5 mm) was performed to analyse the volume, surface area, and untouched walls. Apical transportation and centring capacity were assessed at 3, 5, and 7 mm from the apex. The result obtained showed that, in the apical third, the XP-endo Shaper system was more effective, with a higher increase in volume (22.82%–14.36%) and smaller percentages of untouched wall in the apical segment (23.21%–30.1%) in comparison with the Mtwo system. Relative to the surface area, the study found a percentage of approximately 5.19% for group XP-endo Shaper and 2.28% for group Mtwo. The cantering and apical transportation of the two instruments were similar in all thirds evaluated, and they showed no apical transportation.

Jensen et al. [17] evaluated and compared the preparation capacity of the TRUShape and Vortex Blue systems using micro-CT. Thirty mandibular premolars with oval canals were divided into two groups composed of 15 teeth. Samples were scanned before and after root canal preparation. Parameters such as volume and surface area were evaluated. The root canal preparation with TRUShape obtained an increase in volume of approximately 47.1%, while for Vortex Blue, an increase of 25.35% was obtained. Overall, root canal volumes increased significantly with preparation. However, there were no statistically significant differences between groups. The capacity of TRUShape for preparation in oval root canals was similar to that of Vortex Blue. As regards surface area, for the preparation with TRUShape, a percentage of 24.42% was obtained, whereas with Vortex Blue, it was 13.57%.

The preparation and modelling capacity of BioRace, Reciproc, Self-Adjusting File (SAF), and TRUShape systems was evaluated by Zuolo et al. [18]. Forty mandibular incisors were scanned before and after root canal preparation. Parameters such as percentages of accumulated hard tissue, untouched walls, and dentin removed were evaluated. The canals were combined to create ten groups of four teeth based on similar morphological data such as length, volume, surface area, SMI, and 1 root of each group randomly assigned to one of the four instruments evaluated (*n* = 10). A significantly higher percentage of untouched areas was observed after preparation with the BioRace system (32.38%) when compared with Reciproc (18.95%), SAF (16.08%), and TRUShape (19.20%) systems. Reciproc removed significantly more dentin (4.18%) than BioRace (2.21%), SAF (2.56%) and TRUShape (3.77%). Relative to the percentage of accumulated hard tissue, the study obtained no statistically significant difference.

Versiani et al. [13] evaluated the modelling capacity of the XP-endo Shaper, iRace, and EdgeFile systems by means of micro-CT, in thirty mandibular incisors with long-oval canals. Images captured before and after root canal preparation were evaluated for volume, surface area, SMI, and untouched walls. There were no significant differences in the number of untouched walls (9.42%) for XP-endo Shaper, (8.17%) for iRace and (9.83%) for EdgeFile. For volume, the instruments produced increases of 52.9%, 52.5%, and 52.2%, respectively. The XP-endo Shaper system significantly changed the overall geometry of the root canal to a more conical shape (SMI = 2.59) when compared with the iRace (SMI = 2.34) and EdgeFile (SMI = 2.28) systems. There were also no statistically significant differences in surface area, for which values of 10.8%, 14.2%, and 12.7% were obtained for the XP-endo Shaper, iRace, and EdgeFile instruments, respectively. None of the techniques were able to completely prepare the long-oval canals of mandibular incisors.

Azim et al. [12] evaluated and compared the modelling capacity of the TRUShape and Vortex Blue systems, by using micro-CT. Twenty mandibular incisors with long-oval canals were scanned before and after root canal preparation. Aspects such as the percentage of untouched walls, volume, surface area, and amount of dentin removed were evaluated. XP-endo Shaper performed considerably better in all three thirds (apical, middle, and coronal), with fewer untouched surfaces (38.6%) when compared with Vortex Blue (58.8%). In addition, XP-endo Shaper removed more dentin (1.73–0.98) than Vortex Blue in the coronal and middle thirds. When evaluated for increase in volume, the instruments obtained values of approximately 41.3%∼19.6%. In the evaluation of surface area, there were no significant differences (12.7%–8.3%) for the XP-endo Shaper and Vortex Blue groups, respectively. The analysis allowed the authors to conclude that there was less debris at all levels of the root canal when instrumentation was performed with the XP-endo Shaper.

Three SAF instruments, TRUShape, and XP-endo Shaper were used to evaluate the capacity for cleaning and shaping 33 oval root canals, in a study by Lacerda et al. [19]. Volume, surface area, and untouched walls were the parameters analysed. The distal canals of mandibular molars were digitized by micro-CT before and after preparation, and histological analysis was performed after preparation. The average percentage of untouched walls was 9.85%, 15.88%, and 17.77% for the SAF, TRUShape, and XP-endo Shaper systems, respectively. The instruments performed in 13.48%, 9.39%, and 5.27% of the respective surface areas. The instrument that produced the fewest untouched walls was the SAF with 10.92%, followed by XP-endo Shaper with 17.31% and TRUShape with 17.45%. None of the systems completely prepared the root canal walls.

The TRUShape and Reciproc systems were compared by micro-CT, with respect to the preparation of twenty-six single oval premolar canals, in a study by Guimarães et al. [20]. The samples were divided into two groups for statistical analysis of the increase in root canal volume, surface area, and the number of untouched walls promoted by the two systems. The results showed that the volume increased significantly after preparation with the two systems (23.30%–21.55%) for TRUShape and Reciproc, respectively. When the total length of the root canal was considered, the number of untouched walls was significantly higher for Reciproc (30%) than for TRUShape (24%). Relative to the surface area, the instruments showed a similar performance, with TRUShape attaining 24.11%, and Reciproc, 30.4%. Neither technique was able to completely prepare the oval root canals.  Table 1 shows the synthesis of articles on the preparation of oval and long-oval root canals, included in the literature review.

### 3.1. Comparative Analysis of Studies by Instruments

#### 3.1.1. XP-Endo Shaper

The results found in published articles dealing with instrumentation with the XP-endo Shaper showed different percentages of untouched walls, and these differences could be explained due to methodological variations in the studies. According to Velozo et al. [14], the percentage of untouched walls was 13.08%, in the long-oval canals of mandibular incisors. Versiani et al. [13] and Azim et al. [12] also analysed mandibular incisors with this instrument and found 9.42% and 38.6% of untouched walls, respectively. The samples used in other studies were canines, premolars, and molars. Xavier et al. [16] found 14.19% of untouched walls in canines, Thomas et al. [15] found 52.28% in premolars, and Lacerda et al. [19] obtained 17.31% of untouched walls when preparing distal roots of mandibular molars.

The performance of the instruments in terms of increase in volume after root canal preparation with the XP-endo Shaper was analysed by some authors. Velozo et al. [14] recorded an increase of 107.5% when analysing the preparation of mandibular incisors, while Versiani et al. [13], who also used mandibular incisors, found an increase of 52.9%. The samples used in other studies were canines, premolars, and molars. Thomas et al. [15] found an increase in volume of the root canal of 17.71% in premolars. Lacerda et al. [19], found an increase of 25.41%, when preparing distal roots of mandibular molars, whereas Xavier et al. [16] found an increase of 22.82% in canines.

The increase in surface area was another parameter that could be compared among the works of these authors. The XP-Endo Shaper instrument performed in 27.74% of the surface area in mandibular incisors, when analysed by Velozo et al. [14], while Azim et al. [12] obtained percentages of approximately 12.7% when analysing the same type of teeth. Other authors obtained values in samples with mandibular premolars and molars, and as they were different samples, they were not comparable. The records showed that Thomas et al. [15] obtained 14.23% in premolars, while Lacerda et al. [19] obtained a 5.27% increase in surface area, when preparing distal roots of mandibular molars.

#### 3.1.2. Vortex Blue

The results found in published articles dealing with instrumentation performed with the Vortex Blue instrument showed different percentages of increase in volume. According to Jensen et al. [17], a percentage of 25.35% increase in volume was found in oval canals of premolars, whereas Azim et al. [12] analysed mandibular incisors with this instrument and found a 19.6% increase in volume.

After analysing the increase in surface area throughout the entire extent of the root canal, Azim et al. [12] obtained a value of approximately 8.3% when performing instrumentation of long-oval canals of mandibular incisors with Vortex Blue, whereas Jensen et al. [17] analysed oval canals of premolars; therefore, it was not possible to compare the data with the abovementioned study; however, with the same instrument, they obtained an increase in surface area of approximately 13.57%.

#### 3.1.3. Reciproc

When the percentage of untouched areas and dentin removed by using the Reciproc instrument were analysed in the study by Zuolo et al. [18], they demonstrated that 18.95% of the areas in mandibular incisors remained untouched after preparation and that Reciproc was able to remove 4.18% of the dentin. Guimarães et al. [20] when evaluating this instrument in oval canals of mandibular premolars showed that 30.40% of the areas were not touched after canal preparation with the Reciproc instrument.

#### 3.1.4. TRUShape

The results found in published articles dealing with instrumentation with TRUShape showed different percentages of untouched walls. According to Thomas et al. [15], 55.26% of untouched walls were found in oval canals of premolars. Guimarães et al. [20] also analysed premolars with the TRUShape system and found 24.11% of untouched walls, whereas Lacerda et al. [19] found 17.45% in mandibular molars and Zuolo et al. [18] found 19.20% in mandibular incisors.

The performance of instruments in terms of volume after root canal preparation with the TRUShape instrument was analysed by some authors. According to Thomas et al. [15], they recorded an increase of 19.69% when analysing premolars. Guimarães et al. [20] also analysed premolars and showed evidence of a 23.3% increase in volume, while Jensen et al. [17], when analysing the same type of teeth, found an increase of 10.18%. Another study used mandibular molars, as was the case with Lacerda et al. [19], and obtained a 48.88% increase in volume.

Another parameter that could be compared was the increase in surface area. Three studies analysed this parameter in the preparation of premolars with oval canals using micro-CT. According to Jensen et al. [17], the increase in surface area was 45.08%, characterizing the largest increase among those found by the authors evaluated, namely, Thomas et al. [15] who obtained 14.05% increase in surface area and Guimarães et al. [20] who found 12.34% also in mandibular premolars. Another author used mandibular molars; therefore, their findings could not be compared with studies using other types of teeth as samples, as was the case of Lacerda et al. [19] who found a 9.39% increase in surface area in mandibular molars.

#### 3.1.5. Self-Adjusting File (SAF)

The parameter of untouched areas with instrumented root canals with SAF was evaluated by two authors. Zuolo et al. [18] in their study obtained a percentage of 16.08% of untouched areas in mandibular incisors in preparations performed with the SAF instrument, while Lacerda et al. [19], evaluating the preparation of distal canals of mandibular molars with the same instrument, obtained a percentage of 10.92% of untouched walls considering the entire length of the canal.

## 4. Conclusions

The studies published up to now have demonstrated that no instrument was capable of performing root canal cleaning throughout its entire extent. Despite the strict method of sample selection, using microcomputed tomography, comparison of the results required sample compatibility regarding the anatomy of the root canal, which did not occur in the studies selected. Among the instruments analysed, the XP-endo Shaper had the largest collection of recent studies that allowed the authors to affirm that it had a good performance when the untouched areas were analysed after endodontic preparation.

## Figures and Tables

**Table 1:** Synthesis of articles on the preparation of oval and long-oval root canals, included in the literature review.

Author/year	Samples	Methodology	Results
Velozo et al. [14]	20 long-oval canals (incisors)	To evaluate the performance of the XP-endo® Shaper and ProTaper® Next instruments, in the preparation of long-oval canals, using computed microtomography (micro-CT) technology (*n* = 10)	Root canal preparation led to a significant increase in all the parameters (volume, surface area, structure model index (SMI), and untouched walls) tested in each group (*p* < 0.05). There was no significant difference (*p* > 0.05) in the percentage increase in volume (107.50%–93.13%), surface area (27.74%–29.68%), or intact canal wall (13.08%–11.74%) between XP-S and PTN X4, respectively

Thomas et al. [15]	32 oval canals (premolars)	Microtomographic evaluation of oval canal preparations with WaveOne Gold (G1), TRUShape (G2), EdgeCoil (G3), and XP-3D Shaper (G4) instruments (*n* = 8)	There was no statistically significant difference between the groups for any of the rotary instruments used (P, 0.05). The percentages of untouched walls were as follows: G1: 50.09%, G2: 55.26%, G3: 38.09% E, and G4: 52.28% relative to volume, and the following data were found: G1 22.44%, G2: 19.69%, G3: 27.31% E, and G4: 17.71%; when evaluating the surface area, G1: 19.65%, G2: 14.05%, G3: 24.34% E, and G4: 14.23%

Xavier et al. [16]	40 oval canals (canines)	To evaluate the preparation capacity of XP-Endo Shaper and Mtwo in oval canals, by means of micro-CT (*n* = 20)	None of the systems evaluated could prepare the entire length of the root canal, and there were no statistical differences relative to untouched areas throughout the entire length of the root canal between XP-endo Shaper and Mtwo (14.19% and 12.51%, respectively). When the apical third was analysed, the Shaper was more effective, leading to a lower percentage of unprepared area (22%), when compared with the Mtwo system (30%) (*p* < 0.05)

Jensen et al. [17]	30 oval canals (premolars)	To evaluate the preparation capacity of TRUShape and Vortex Blue instruments in oval canals, by using micro-CT (*n* = 15)	The preparation capacity of TRUShape (45.08%) in oval canals was similar to that of Vortex Blue (42.99%). TRUShape significantly improved the surface treatment. No file system was capable of coming in contact with or completely preparing the entire surface of the root canal in oval canals

Zuolo et al. [18]	40 oval canals (mandibular incisors)	Use microtomography to evaluate the shaping capacity of the systems. BioRace, Reciproc, Self-Adjusting File (SAF), and TRUShape systems (*n* = 10)	The preparation techniques did not affect the percentage of hard tissue residues that accumulated (*p*=0.126). The percentage of untouched canal areas was significantly higher for BioRace (32.38%), and Reciproc (18.95%) with the results of the SAF system (16.08%) showing the least untouched area (*p* < 0.05). The Reciproc system removed significantly more dentin (4.18%) in comparison with the BioRace (2.21%) and SAF (2.56%) systems (*p* < 0.05)

Versiani et al. [13]	30 long-oval canals (incisors)	Evaluation of the root canal preparation performed with the XP-endo Shaper, iRace (R1, R2 and R3), and EdgeFile (X1 and X7) by means of micro-CT (*n* = 10)	The XP-Endo Shaper, iRace, and Edgefile showed similar shaping capacity. The mean value of untouched walls was 9.42% for XP-Endo Shaper, 8.17% for iRace, and 9.83% for EdgeFile. There was no significant difference in the quantity of untouched walls among the groups analysed

Azim et al. [12]	20 long-oval canals (incisors)	To evaluate the shaping capacity of XP-endo Shaper (G1) and compare the values with the results of Vortex Blue (G2) by means of micro-CT (*n* = 10)	XP-endo Shaper had significantly fewer untouched walls (38.6%) in comparison with Vortex Blue (58.8%). After preparation, the volume values were 41.3% (G1) and 19.6% (G2). The surface area found in G1 was 12.7% and for G2, 8.3%. Therefore, XP-Endo Shaper was capable of preparing and touching more walls than Vortex Blue

Lacerda et al. [19]	33 oval canals (mandibular molars)	To evaluate the cleaning and preparation capacity of three instrumentation systems: Self-Adjusting File (G1), TRUShape (G2), and XP-endo Shaper (G3) (*n* = 11)	The mean number of untouched areas after preparation with G1, G2, and G3 were 10.92%, 17.45%, and 17.31%, respectively. The surface area was also evaluated, showing G1: 13.48%, G2: 9.39% E, and G3: 5.27%. The volume found was 63.11% for G1, 48.88% for G2, and 25.41% for G3

Guimarães et al. [20]	26 oval canals (mandibular premolars)	To evaluate the preparation capacity of TRUShape and Reciproc in oval canals, by means of micro-CT (*n* = 13)	The systems behaved in a similar manner in relation to the increase in root canal volume (23.30%–21.55%) and surface area (12.34%–13.74%), respectively. For TRUshape, the unprepared surface area was 24.11%, in comparison with 30.40% for Reciproc

## Data Availability

No data were used to support the study.
